# Exploring the Extent of Phosphorus and Heavy Metal Uptake by Single Cells of *Saccharomyces cerevisiae* and Their Effects on Intrinsic Elements by SC-ICP-TOF-MS

**DOI:** 10.3389/fmicb.2022.870931

**Published:** 2022-04-25

**Authors:** Wen Qin, Hans-Joachim Stärk, Susann Müller, Thorsten Reemtsma

**Affiliations:** ^1^Department of Analytical Chemistry, Helmholtz Centre for Environmental Research – UFZ, Leipzig, Germany; ^2^Department of Environmental Microbiology, Helmholtz Centre for Environmental Research – UFZ, Leipzig, Germany; ^3^Institute of Analytical Chemistry, University of Leipzig, Leipzig, Germany

**Keywords:** elemental distribution, transition metals, phosphorus, absorption, toxicity, adsorption

## Abstract

The effect of six heavy metals, namely, silver (Ag), lead (Pb), palladium (Pd), copper (Cu), nickel (Ni), and chromium (Cr), on phosphorus (P) uptake by yeast was investigated by single-cell analysis using inductively coupled plasma time-of-flight mass spectrometry (SC-ICP-TOF-MS). It was found that the P content in cells with 1.55 g L^–1^ P feeding after P starvation was increased by ∼70% compared to control cells. Heavy metals at 10 ppm, except Cu, had a negative impact on P accumulation by cells. Pd reduced the P content by 26% in single cells compared to control cells. Metal uptake was strongest for Ag and Pd (0.7 × 10^–12^ L cell^–1^) and weakest for Cr (0.05 × 10^–12^ L cell^–1^). Exposure to Cr markedly reduced (−50%) Mg in cells and had the greatest impact on the intrinsic element composition. The SC-ICP-TOF-MS shows the diversity of elemental content in single cells: for example, the P content under standard conditions varied between 12.4 and 890 fg cell^–1^. This technique allows studying both the uptake of elements and sublethal effects on physiology at a single-cell level.

## Introduction

P. resources are essential for human life and development and are indispensable in agriculture and the chemical industry. Currently, most of the P resource demands the mining of natural phosphate rock (Ohtake and Tsuneda, 2019). This ore resource is non-renewable and has limited reserves worldwide, which means that it cannot guarantee the increasing demand for P. However, it is estimated that up to 2.4 × 10^9^kg of P per year will enter surface water globally by 2050 (Van Puijenbroek et al., 2019). This not only wastes valuable resources but may also pollute the environment (e.g., causing eutrophication). Therefore, the enrichment, recovery, and reuse of P from wastewater are becoming particularly important and urgent. The main methods of P recovery from wastewater are physical, biological, and chemical approaches (Carrillo et al., 2020; Vučić and Müller, 2021). Yeast cells are considered to be a by-product of some industrial sectors, and it has been studied in the past to reuse them to accumulate P (Beever and Burns, 1981; Watanabe et al., 2008; Christ and Blank, 2019). Several methods, such as light and fluorescence microscopy combined with specific staining and electron microscopy, have been reported for the analysis of P accumulated in phosphate accumulating organisms (PAOs) (Tarayre et al., 2016). However, it remains a challenge to accurately analyze the details of P content and distribution at the single-cell level, and thus, a new approach is urgently needed in this field.

Wastewaters from households as well as from industry usually contain various heavy metals, such as chromium (Cr), copper (Cu), and lead (Pb) (Fu and Wang, 2011; Azimi et al., 2017). These metals may also be enriched if the yeast is employed for P recovery from wastewater. This would affect the applicability of yeast cells for P accumulation (Vohla et al., 2011). Moreover, heavy metals may exert toxic effects on yeast (Soares et al., 2003), which could influence the physiology of the cell and, thus, its ability to accumulate P. However, it is still unclear to what extent heavy metals in wastewater affect the bioaccumulation of P and whether or not the physiological states of yeast cells would be affected.

The conventional method for analyzing the elemental content of cells commonly referred to as bulk analysis involves digesting a large number of cells at once by acid digestion to obtain an average result representing the entire cell population. However, this average value cannot disclose the differences between individual cells, which is known as cell heterogeneity and caused by different factors, such as cell cycle phases, cell age, gene expression, and physiological cell states (Taniguchi et al., 2010; Buettner et al., 2015). Analyzing this difference can help to solve many challenges, such as the phenomenon that individual cells respond differently to drugs (Klepárník and Foret, 2013; Corte Rodríguez et al., 2017). For this purpose, many techniques have been developed, including flow cytometry, laser ablation electrospray ionization mass spectrometry (LA-ESI-MS), and others (Wang and Bodovitz, 2010; Heath et al., 2016).

Recently, single cell-inductively coupled plasma-mass spectrometry (SC-ICP-MS) has been developed to reveal the heterogeneity of single cells by detecting elemental information from the cells (Mavrakis et al., 2019; Theiner et al., 2020; de Vega et al., 2021). There are more discoveries at the single-cell level reported employing this method. For instance, *S. cerevisiae* was found to exhibit a broader distribution of elements (e.g., Mg, P, and K) in the exponential growth phase compared with the stationary phase (Qin et al., 2021a). Furthermore, the elemental distributions of iron (Fe), zinc (Zn), Cu, and manganese (Mn) were shown not to be identical in three different human cells, namely, HeLa, A549, and 16HBE cells through cell suspension analysis (Wang et al., 2015). Additionally, the interaction of silver nanoparticles with yeast cells was recently studied using SC-ICP-MS (Rasmussen et al., 2022).

If a time-of-flight mass spectrometer is applied to SC-ICP-MS, the method is referred to as SC-ICP-TOF-MS. In contrast with a quadrupole detector, a time-of-flight mass spectrometer allows recording of all elements that occur above their limit of quantification in parallel. For instance, it was found that there is a good correlation between the content of P and Zn in the THP-1 (human cell line derived from monocytic leukemia) cell line but not between P and Mg (Lohr et al., 2019). By measuring the element fingerprint, including Mg, silicon (Si), P, Fe, and Zn, different algae cell species have been distinguished (von der Au et al., 2020). This opens up the possibility to determine cell species and cell strains based on the patterns of elemental content in the cells using the SC-ICP-TOF-MS. Therefore, it should be a potent and appropriate approach for detecting the P contents and distributions in cell populations after P accumulation and the effects of heavy metals on intrinsic element compositions in single yeast cells.

In this study, the ability of *S. cerevisiae* to accumulate P was analyzed at the single-cell level using the SC-ICP-TOF-MS. Different types and concentrations of heavy metals were studied for their effects on P accumulation and the distribution of heavy metal elements that interacted with individual cells. The study contributes to the understanding of the effects of heavy metal types and concentrations on biological P recovery by means of yeast cells, thus supporting the new development of industrial-scale biological recovery strategies, which may benefit wastewater treatments.

## Materials and Methods

### Chemicals

Element standards were purchased from Merck (Darmstadt, Germany) with a concentration of 0.5 mol L^–1^ and quality level of MQ 300: lead [Pb(NO_3_)_2_], silver (AgNO_3_), palladium [Pd(NO_3_)_2_], nickel [Ni(NO_3_)_2_], chromium [Cr(NO_3_)_3_], and copper [Cu(NO_3_)_2_]. The 60-nm standard gold nanoparticle was provided by BBI Solution Company from Crumlin in Wales, the United Kingdom. According to the manufacturer, the mean size range of nanoparticles was 57.0–63.0 nm. The number density of particles was 2.60 × 10^10^ particles ml^–1^. To avoid particle aggregation, the nanoparticle stock solution was first sonicated for at least 5 min using the SONOREX super RK 31 from Bandelin (Berlin, Germany), and then diluted to the required concentrations. Ultrapure water (Milli-Q water) in this study was constantly provided by the Milli-Q IQ 7003 system from Merck (Darmstadt, Germany). EQ™ Four Element Calibration Beads (hereafter, polystyrene beads) with a particle number concentration of 3.3 × 10^5^ ml^–1^, which contained four elements, namely, cerium (^140/142^Ce), europium (^151/153^Eu), holmium (^165^Ho), and lutetium (^175/176^Lu), were purchased from Fluidigm (San Francisco, CA, United States).

### Yeast Cell Cultivation and Preparation

The cultivation of *S. cerevisiae* was performed on Schatzmann medium (Schatzmann, 1975). The P concentration was adapted to meet different experimental setups, namely, 0.434 g L^–1^ (standard Schatzmann medium), 0 g L^–1^ (P-free medium), and 1.55 g L^–1^ (P-excess medium). The other constituents of the medium remained unchanged. The medium composition is described in Supplementary Table 1 and a previous paper (Qin et al., 2021a).

*S. cerevisiae* H155 was used for the study and was obtained from strain collection at the Helmholtz Centre for Environmental Research in Leipzig. To prepare yeast cells for the experiments, they were incubated in a 500-ml Erlenmeyer flask with 100 ml of the standard medium at a starting optical density (OD_600_, λ = 0.5 cm) of 0.1 (approximately 10^6^ cells ml^–1^) and grown at 125 rpm for 48 h at 30°C. Then, 100 ml of cell suspension was centrifuged at 6,000 × *g* for 10 min at 4°C by Eppendorf 5810 centrifuge (Munich, Germany). The supernatant was carefully discharged, and the cell pellets were kept and processed immediately in the following steps.

For SC-ICP-TOF-MS analysis, the cells were washed two times with ultrapure water using the Heraeus Fresco 21 from Thermo Fisher (Darmstadt, Germany) at 6,000 × *g* for 10 min. After centrifugation, the supernatant was removed to reduce noise signals from the solution. The cells were resuspended in ultrapure water at a cell density of approximately 5 × 10^5^ cells ml^–1^ directly before the single-cell analysis.

A disposable hemocytometer, C-Chip (NanoEntek, Seoul, South Korea), was applied for cell density measurements. The microscope Leica DM 5500 B from Leica Microsystems (Wetzlar, Germany) was used for cell observation.

### Incubation Conditions of P Depletion and P Excess

Before applying P depletion or P excess conditions to the cells, the old media (standard Schatzmann medium or P-free medium) were removed by centrifugation using the conditions as mentioned above. First, the old media were removed up to approximately 0.5 ml using a pipette, and then the remaining was carefully removed with a syringe to ensure that the remaining P is negligible. Then the cells were resuspended in either 100 ml fresh P-free medium or P-excess medium and incubated under the same conditions (30°C, 125 rpm) for 4 or 2 h.

### Heavy Metal Exposure to Cells During the P Excess Conditions

To study the influence of heavy metal exposure during the P accumulation process by single cells of *S. cerevisiae*, different amounts of concentrated heavy metal standard solutions were added to the culture medium to eventually reach the required exposure concentrations of 0.1, 1, and 10 ppm. The incubation was performed under the same conditions as mentioned above. The cells of the strain used in this experiment, *S. cerevisiae* H155, were always treated with a 4 h P starvation period before being supplied with exceeding P concentrations for 2 h in the heavy metal exposure during P accumulation experiments due to the advantages of the P starvation procedure (refer to the section “Results and Discussion”).

### Cell Viability Analysis

After heavy metal exposure, cells were harvested and resuspended in PBS buffer solution. Methylene blue was used for the determination of cell viability. This molecule can freely pass through the cell membrane and enter the dead cells, turning them blue. On the contrary, living cells subjected to this treatment will be colorless due to the presence of cell membrane selectivity. Before use, methylene blue was freshly dissolved in a 2% dehydrate sodium citrate solution at a concentration of 0.1 mg ml^–1^. In detail, 0.5 ml of cell suspension with a cell density of 10^8^ cells ml^–1^ was mixed well with 0.5 ml of methylene blue solution and incubated at room temperature for 5 min (Kwolek-Mirek and Zadrag-Tecza, 2014). The cell viability was analyzed after heavy metal exposure using an optical microscope. The observation areas were randomly chosen, and at least 77 (mean 114) individual cells in each cell photograph (3 photos per sample) were analyzed for their viability states. For cell staining, methylene blue was obtained from Sigma-Aldrich (Darmstadt, Germany).

### SC-ICP-TOF-MS and the Parameters

For single-cell analysis, the ICP-TOF-MS model, called “icpTOF-R” from Tofwerk (Thun, Switzerland), was used with typical parameters, including sample inlet speed, nebulizer gas flow, and target isotopes, as listed in Supplementary Table 2. The ICP-TOF-MS was maintained daily according to the instructions. The tuning solution, which contained standard elements uranium, indium, and cobalt at the concentration of 1 ppb in 1% ultrapure HNO_3_ solution, was used for auto-tuning directly before measurements. In addition, ultrapure water was injected into the inlet system at least half an hour before the cell measurement for thorough cleaning. Between each cell measurement using the SC-ICP-TOF-MS method, 1% HNO_3_ and ultrapure water were separately introduced to wash and clean the sample transportation system for at least 3 min. The integration time was set to 3 ms per sample (Qin et al., 2021a), and the measurement mode was selected as time-resolved. In this study, the cell suspension with a cell number concentration of 5 × 10^5^ cells ml^–1^ was delivered at an injection speed of 0.33 ml min^–1^ for measurement (Qin et al., 2021b). For the SC-ICP-TOF-MS analysis of each cell sample, at least 427 individual cells were analyzed (Supplementary Table 3).

To determine liquid transport efficiency (ϕ_liquid_) through the sample inlet system of SC-ICP-TOF-MS, a waste collection method (approach A) was applied. A volume of 10 ml of water was introduced into the system and the waste was collected until no liquid droplets were detected. The following equation was used for the calculation of this value (m_waste_ is the mass of collected water mass):


(1) $$\phi_{l\,i\,q\,u\,i\,d}=\frac{10\,g-m_{w\,a\,s\,t\,e}}{10\,g}\times 100\%\;$$

To determine the transport efficiency of cells, nanoparticles, and polystyrene beads (ϕ_cell/particle_), the number of signals counted from the SC-ICP-TOF-MS system during 1 min (n _signal_) was compared to the number of items (cells, beads, and particles), which were fed into the system in this period of time (approach B). The following Eq. 2 was used:


(2) $$\phi_{c\,e\,l\,l/p\,a\,r\,t\,i\,c\,l\,e}=\frac{n_{s\,i\,g\,n\,a\,l}}{\delta_{c\,e\,l\,l/p\,a\,r\,t\,i\,c\,l\,e}\times \nu \times 1\,m\,i\,n}\times 100\%$$

In the equation above, δ_cell/particle_ is the density of cell or particle suspension (number/ml) and υ represents the sample inlet speed (ml/min).

### Data Analysis

Each cell sample was injected into the ICP-TOF-MS and all elements in a single cell generated signals proportional to their amounts. The software Tofware^1^ from Tofwerk (Thun, Switzerland) was used to distinguish the cell signals from the background. Elemental signals and mass data of single cells were further analyzed and visualized using Excel and OriginPro software.

## Results and Discussion

### Cell Signal and Transport Efficiency

**FIGURE 1:**
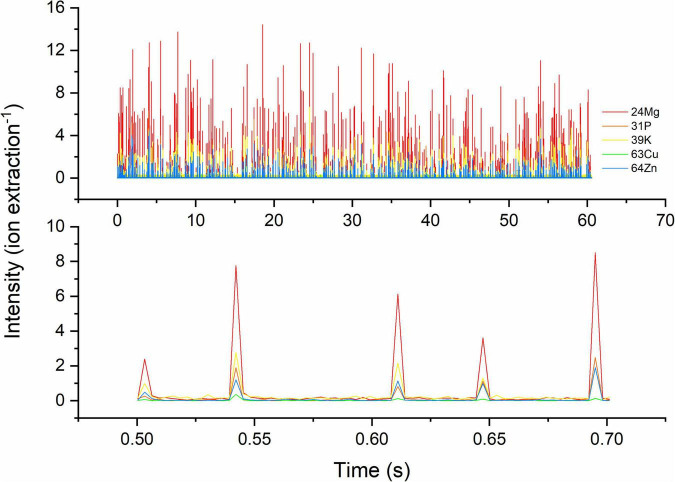
Time-resolved transient signals of five target elements of single cells of *S. cerevisiae*. Target isotopes are ^24^Mg, ^31^P, ^39^K, ^63^Cu, and ^64^Zn. Above: signals within 60 s; below: a zoomed-in section of transient signal, 0.50–0.70 s showing the co-occurrence of 4–5 of these elements. The signal intensity is dependent on absolute mass in single cells and the response of isotope in TOF-MS.

The use of a TOF mass spectrometer allows recording of the elemental signals of five intrinsic elements (Mg, P, K, Cu, and Zn) in single *S. cerevisiae* cells simultaneously (Figure 1). All target atoms in one cell produce signal peaks at the same integration time, and their intensity is proportional to their absolute amount. Other intrinsic elements could not be detected in this study because of high backgrounds or extremely low absolute masses in a single cell.

The transport efficiency describes the proportion of an element delivered to the ICP-MS system that makes its way through the nebulizing system and actually arrives in the ICP. For metal cations in solution, this is assessed by measuring the loss of solution through the waste flow of the nebulizer. On this basis, the transport efficiency (ϕ_liquid_) for dissolved metal cations was 8.05% (Table 1). However, it is well known that the waste collection method overestimates the transport efficiency of ICP-MS (Pace et al., 2011). The transport efficiency for particles in suspension may be lower than for a solution as particles may deposit on the inner walls of the tubing or the spray chamber. Based on the total number of particles delivered to the analytical system and the number of events recorded by the ICP-MS, the transport efficiency recorded for gold nanoparticles was 5.56%. It was, in fact, lower than that for the solution (Table 1). For polystyrene beads labeled with Ce, Eu, Ho, and Lu, the transport efficiency was even lower (Table 1).

**TABLE 1:** Transport efficiency (ϕ) for Milli-Q water (by approach A), 60 nm gold nanoparticle suspension, 4-element polystyrene beads, and *S. cerevisiae* cell suspension (by approach B).

	Milli-Q water	60 nm AuNP	Polystyrene beads	*S. cerevisiae* cells
Diameter	–	0.06 μm	2.5 μm	3 – 15 μm (Qin et al., 2021a)
Test 1	8.10%	5.55%	1.42%	0.24%
Test 2	8.16%	5.72%	1.75%	0.18%
Test 3	7.90%	5.40%	1.49%	0.21%
Average ± SD	8.05 ± 0.13%	5.56 ± 0.16%	1.55 ± 0.18%	0.21 ± 0.03%

*SD, standard deviation.*

The SC-ICP-MS method can analyze hundreds of cells in a few minutes (Theiner et al., 2020). For *S. cerevisiae* cells in this study, the transport efficiency (ϕ_cell_) was 0.21% (Table 1). This value agrees with literature values, all less than 1% (Meyer et al., 2018; Qin et al., 2021a). The data in Table 1 suggest that the transport efficiency of particles decreases with increasing particle mass and size. Additionally, polysaccharides and protein structures on cell surfaces may further reduce the transport efficiency of cells by increasing interactions with the inner walls of the capillary tubes and spray chambers.

### P Content in Single Cells of *S. cerevisiae*

**FIGURE 2:**
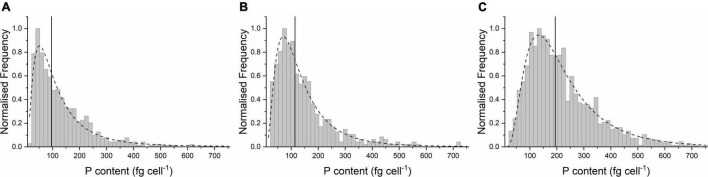
The elemental distribution of P of single cells of *S. cerevisiae* under conditions. **(A)** Control cells at 0.434 g L^–1^ P, **(B)** cells at 1.55 g L^–1^ P for 2 h, and **(C)** cells at 0 g L^–1^ P for 4 h before the incubation at 1.55 g L^–1^ P for 2 h. Each distribution is separately fitted by a lognormal function (dash lines in the graph). The distribution centers (Xc) are marked by vertical lines.

After being incubated with a concentration of 0.434 g L^–1^ P of the standard medium, the mean P content in the yeast was determined to be 128 fg cell^–1^. Single-cell analysis showed that the P content of single cells varies by more than one order of magnitude, from 20 fg cell^–1^ to > 500 fg cell^–1^ (25–75 percentile: 49–173 fg cell^–1^) (Figure 2A and Table 2). When cells were incubated with 1.55 g L^–1^ P (P-excess medium) for 2 h, the P content increased only slightly (Figure 2B), with a mean of 140 fg cell^–1^ (+9.4% compared to the control cells) (Table 2). However, if kept under P starvation for 4 h before being exposed to 1.55 g L^–1^ of P, the P content of the *S. cerevisiae* cells increased drastically (Figure 2C). The lognormal distribution center increased to 194 fg cell^–1^ and the mean concentration to 218 fg cell^–1^ (Table 2), about 70 and 56% higher than the results from the cells without starvation.

**TABLE 2:** P content in single cells of *S. cerevisiae* after three different P exposures.

Parameter	0.434 g L^–1^ P	1.55 g L^–1^ P	0 g L^–1^ P, then 1.55 g L^–1^ P
Minimum (fg cell^–1^)	12.4	15.2	15.5
Maximum (fg cell^–1^)	890	710	1.17 × 10^3^
Mean (fg cell^–1^)	128	140	218
Median (fg cell^–1^)	94.5	107	184
Xc (fg cell^–1^)	96.0	114	194
25 – 75 percentile (fg cell^–1^)	49.2 – 173	64.5 – 173	119 – 282

*A box plot of these three cell samples can be found in Supplementary Figure 1.*

*Xc is the center of lognormal distribution in Figure 2.*

These findings at the single-cell level agree with literature reports that a low-P environment triggers the expression of the PHO gene (responsible for an enhanced P uptake) and the use of the stored P sources to fulfill cell growth needs (Kaffman et al., 1998; Lau et al., 1998; Ogawa et al., 2000). If then placed in a medium containing P, the higher PHO level leads to enhanced P uptake; this phenomenon is known as polyP hyperaccumulation (Liss and Langen, 1962; Lawry and Jensen, 1979). Previously, this yeast strain was shown to exhibit a wider distribution of P content during its exponential growth phase compared to its stationary phase; this was interpreted as reflecting the larger diversity in the development of the individual cells in this period of rapid development (Qin et al., 2021a). The wider distribution of P content in the 1.55 g L^–1^ P-excess medium may indicate that this cell culture developed more rapidly and had already entered its exponentially growing phase when analyzed; in agreement with this, the microscopic pictures revealed more budding cell structures on the cell surface (Supplementary Figure 2).

### Influence of Heavy Metals on the P Accumulation Capabilities of Individual Cells of *S. cerevisiae*

**FIGURE 3:**
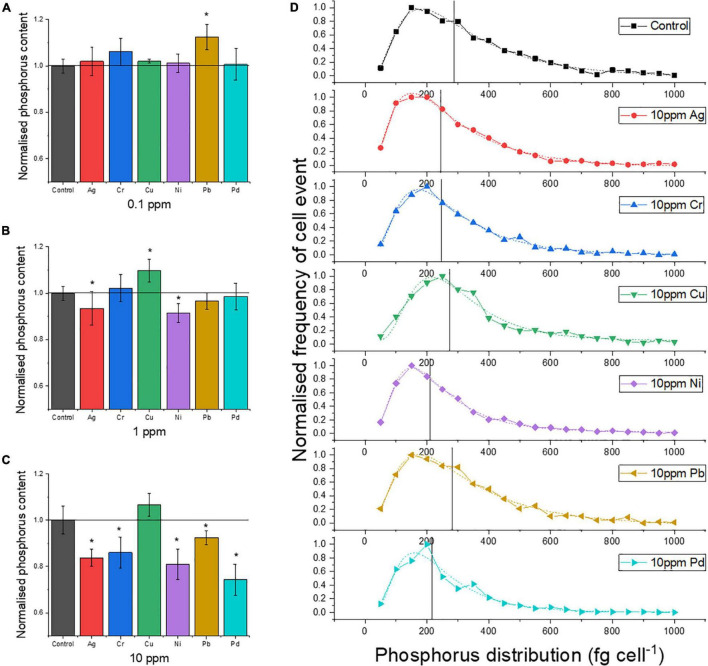
Mean P content of single cells normalized to the control, after exposure to heavy metals with concentrations of **(A)** 0.1, **(B)** 1, and **(C)** 10 ppm. **(D)** P distribution of single cells at 10 ppm metal exposure (dash lines represent lognormal distribution and the distribution centers; Xc are marked by vertical lines in the graph). Asterisk mark means the P content is significantly different from the control group, *p* < 0.05.

It was tested to what extent exposure of yeast cells to one of six heavy metals (Ag, Pb, Pd, Cu, Ni, and Cr) affected their P uptake in the P-excess medium by yeast cells after the pretreatment of P starvation. The heavy metal Pb was found to support the P accumulation (+12%) at 0.1 ppm concentrations (Figure 3A), while all other metals had no significant effect at this concentration. With increasing exposure concentration (1 ppm, 10 ppm), the effects on P uptake became more pronounced (Figures 3B,C): Ag, Cr, Pb, and Pd led to a decreasing P uptake, while Cu promoted P uptake. The strongest suppression was found for Pd, with a reduction of about 30% in P uptake at 10 ppm compared to the control.

The frequency distribution of the P content in the individual cells (Figure 3D) provides further insight into the effects of the metals: the lower average P content caused by some of the heavy metal exposure is not due to the shift in the frequency maximum to a lower value. Instead, the frequency of cells with higher P content is relatively lower, as seen for Pd and Ni, for which the center of the P distribution (Xc) is clearly lower than for the control. For Ag and Cr, this shift is weaker (Figure 3D), as is the decrease in the mean P content (Figure 3C). For Cu, the exposure resulted in a higher mean P content (+7%) because the frequency maximum was clearly shifted to approximately 250 fg cell^–1^ compared to 150 fg cell^–1^ for the control, while the Xc value hardly shifted (Figure 3D).

This drop in the mean P content upon exposure to five of the metals was not due to a loss of available free P by precipitation: the P concentration (g L^–1^ range) was at least two orders of magnitude higher than that of the metals (mg L^–1^ range).

Previously, *S. cerevisiae* cells were found to be able to release intracellular P as a response to heavy metal stress by forming precipitates on the cell wall, and this process is known as “self-protection” (Zheng et al., 2017). Thus, the lower P content of the yeast cell exposure to six of the metals may be a consequence of such an excretion of P from the cells. Moreover, heavy metal ions may have an inhibitory effect on cell growth (Chen and Wang, 2007), causing cells to remain smaller, with a correspondingly lower P content per cell. Heavy metals may also affect the gene expression of *S. cerevisiae* (Yasokawa et al., 2008; Hosiner et al., 2014), which may interfere with enzymes involved in P uptake and polyphosphate synthesis, and ultimately lead to reduced P uptake.

On the contrary, Cu shows a promoting effect on P uptake. This may be due to the fact that Cu is an indispensable element in cells and an important part of many enzymes (Gralla et al., 1991; De Freitas et al., 2003); a good supply of Cu may promote certain biochemical activities and, eventually, P uptake. It may also have supported cell growth: larger cells would exhibit a higher P content per cell, without an increase in their P concentrations.

### Uptake of Heavy Metals by Individual Cells of *S. cerevisiae*

With the help of SC-ICP-TOF-MS, the uptake of heavy metal ions by single yeast cells depending on the exposure concentrations was studied for the excess P concentrations (1.55 g L^–1^).

At low metal exposure (0.1 ppm), Cr, Ni, and Pb could not be detected from any of the individual cells (Supplementary Figure 3) above the element-specific Limit of detections (LODs) of 0.065, 0.115, and 0.002 fg cell^–1^, respectively (Table 3). Ag and Pd were found in approximately 20% of the single cells (Supplementary Figure 3), and their average cellular contents from the detected cells with heavy metals were 0.16 and 0.18 fg cell^–1^, respectively (Table 3). *S. cerevisiae* was previously used for the biosorption of these two heavy metals, suggesting a favorable affinity toward Ag and Pd (Simmons and Singleton, 1996; Godlewska-Żyłkiewicz, 2003). As an intrinsic element, Cu should be present in all individual cells. It is one important cofactor of many enzymes, including cytochrome c oxidase (complex IV), that participate in many biochemical processes of cells (Linder, 2013). Since the P excess medium already contained about 63.5 μg L^–1^ of Cu (Supplementary Table 1), the total concentration of Cu in the medium was approximately 0.16 mg L^–1^ after adding 0.1 mg L^–1^ Cu. From 97% of the individual cells, Cu was determined with a mean elemental content of 0.98 fg cell^–1^ (Supplementary Figure 4 and Table 3), which is comparable to the control sample (1.04 fg cell^–1^).

**TABLE 3:** Mean metal content of single cells determined by SC-ICP-TOF-MS (from three measurements of the same cell suspension).

Heavy metal	0.1 ppm exposure			1 ppm exposure			10 ppm exposure		
			
	From cells detected with heavy metal (fg cell^–1^)	From all cells		From cells detected with heavy metal (fg cell^–1^)	From all cells		From cells detected with heavy metal (fg cell^–1^)	From all cells	
						
		Mean metal content (fg cell^–1^)	Ratio of cellular content and exposure concentration (10^–12^ L cell^–1^)		Mean metal content (fg cell^–1^)	Ratio of cellular content and exposure concentration (10^–12^ L cell^–1^)		Mean metal content (fg cell^–1^)	Ratio of cellular content and exposure concentration (10^–12^ L cell^–1^)
Pb	<LOD (0.002)	<LOD (0.002)	–	0.05 ± 0.03	0.01 ± 0.004	0.01	4.08 ± 1.67	3.14 ± 1.20	0.31
Ag	0.16 ± 0.03	0.03 ± 0.01	0.3	0.49 ± 0.05	0.39 ± 0.05	0.39	7.87 ± 0.45	7.62 ± 0.41	0.76
Pd	0.18 ± 0.01	0.04 ± 0.01	0.4	0.41 ± 0.02	0.39 ± 0.02	0.39	7.40 ± 0.57	7.31 ± 0.64	0.73
Ni	<LOD (0.115)	<LOD (0.115)	–	<LOD (0.115)	<LOD (0.115)	–	2.59 ± 0.07	2.56 ± 0.05	0.26
Cr	<LOD (0.065)	<LOD (0.065)	–	<LOD (0.063)	<LOD (0.063)	–	0.53 ± 0.02	0.49 ± 0.02	0.05
Cu	0.98 ± 0.10	0.95 ± 0.10	9.5	1.40 ± 0.09	1.37 ± 0.09	1.37	3.81 ± 0.08	3.73 ± 0.14	0.37

*The values in this table represent the mean elemental contents of the single cells only detected with heavy metal and of all single cells. The ratio (unit: 10^–12^ L cell^–1^) of cellular content and exposure concentration for each heavy metal was also represented.*

When the heavy metal exposure concentration rose to 1 ppm, Pb, with a mean value of ∼0.05 fg cell^–1^ (Table 3), was detected in 19% of the single cells (Supplementary Figure 3), while Ni and Cr were still not detected above their LODs. *S. cerevisiae* cells were reported to possess a higher biosorptive capacity for Pb than for Ni and Cr (Özer and Özer, 2003), which agrees well with these results. The percentage of cells with measurable Ag and Pd contents increased to 78 and 96% at 1 ppm exposure, respectively (Supplementary Figure 3).

At an exposure concentration of 10 ppm, all six target metals were determined from the single yeast cells. Among them, Ag, Pd, and Ni were detected in almost all individual cells above their LODs (Supplementary Figure 3). Pb and Cr were detected in 78 and 92% of the cells, with a mean cellular content of 4.08 and 0.53 fg cell^–1^ from these cells (Table 3 and Supplementary Figure 3). At an exposure level of 10 ppm, the mean cellular Cu content rises to 3.81 fg cell^–1^, which is almost four times that of the control group, and Cu was detected in 98% of single cells (Supplementary Figure 4).

Studies have shown that the surface of yeast cells is negatively charged, which promotes the interaction with positively charged metal ions from the solution (Kordialik-Bogacka, 2011). The uptake of heavy metals consists of two phases: first, metal ions are rapidly adsorbed on the cell surface through interaction with metal-functional groups; and second, these metals on the cell surface are transported into the cell through the cell wall and cell membrane, a process which is energy-dependent and slower than the adsorption step (Wang and Chen, 2006). In the 2-h time frame of this study, a part of metal ions was likely adsorbed on the cell surface, while another part entered the cell. It was reported that *S. cerevisiae* cells needed 5 s of surface binding and 2.5 h of active transportation to take up Cu ions into the cell interior (Kapoor and Viraraghavan, 1995). For Ni uptake, the cells needed approximately 30 min and 24 h for both uptake phases, respectively (Ferraz et al., 2004). The SC-ICP-MS cannot distinguish between adsorbed and internalized metal ions; the sum of both is called “cellular content” in this study.

For the two metals that were detectable at all three exposure concentrations (Ag and Pd), the ratio of the mean cellular content to the exposure concentration was fairly linear, increasing only from 0.3 to 0.7 × 10^–12^ L cell^–1^. Thus, the uptake was proportional to the exposure concentration. Based on these ratios at 10 ppm, at which it could be determined for each of the metals, the extent of their uptake by yeast cells varied by one order of magnitude: Ag ≈ Pd (0.7 × 10^–12^ L cell^–1^) > Pb > Ni > Cr (0.05 × 10^–12^ L cell^–1^). Again, Cu showed a different behavior: here, the ratio of the cellular Cu content to the exposure concentration of Cu decreased by more than one order of magnitude, from 9.5 (at 0.1 ppm) to 0.37 × 10^–12^ L cell^–1^ (at 10 ppm). This suggests that yeast cells can actively regulate their Cu content.

As it determines the metal mass of single cells, the SC-ICP-TOF-MS provides a more differentiated view on metal content. For instance, at 0.1 ppm exposure concentration, the average Ag mass of single cells in which Ag could be detected was 0.16 fg cell^–1^, which is much higher than the average cellular Ag content of 0.03 fg cell^–1^, which includes also the cells with non-detects (the individual cells with lower contents than LODs). Such information may be crucial for studying the toxicity of low-concentration heavy elements to microorganisms.

**FIGURE 4:**
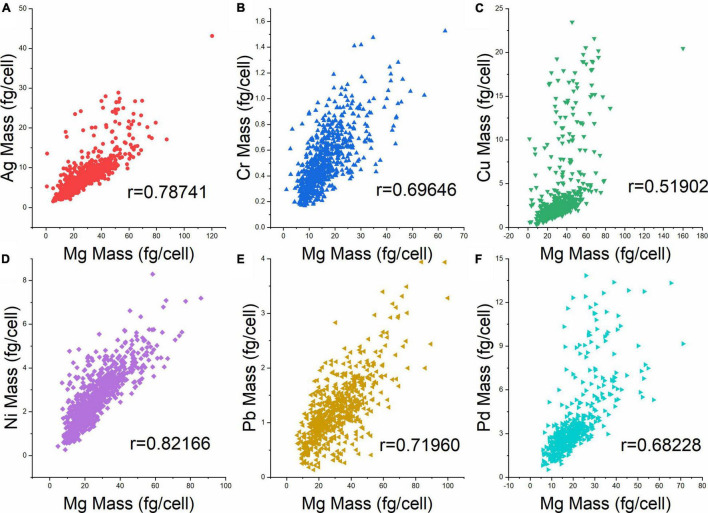
Cellular heavy metal content vs. Mg content (fg cell^–1^) at 10 ppm metal exposure. **(A)** Ag, **(B)** Cr, **(C)** Cu, **(D)** Ni, **(E)** Pb, and **(F)** Pd. R denotes the Pearson’s correlation coefficient between the respective metal and Mg.

Previously, the intrinsic element Mg in yeast cells was used to indicate the volume of individual cells (Qin et al., 2021b). Therefore, the metal mass per cell was plotted against the Mg mass per cell to obtain information on the distribution of heavy metal elements with regard to cell size (Figures 4A–F). While all six metals showed a trend of increasing mass per cell with increasing size (Mg mass per cell), the correlations were not equally strong. The Pearson’s correlation coefficient (r) was highest for Ag and Ni (0.79 and 0.82), followed by Cr, Pd, Pb, and Cu (0.52). Given that also in these experiments, Mg mass was a suitable proxy of cell volume, these data show that cells of similar volume can markedly differ in their heavy metal contents. Obviously, the ability to analyze cellular metal contents from single cells by SC-ICP-TOF-MS provides a more differentiated view of the cell population. This may also be useful to study toxicology and effects of heavy metals on cell populations and on individual cells from a more in-depth and comprehensive perspective.

### The Effect of Heavy Metal Exposure to Other Intrinsic Elements in Single Cells of *S. cerevisiae*

The option to detect the mass of several elements in one cell by SC-ICP-TOF-MS currently allows checking if the uptake of one heavy metal into a cell affects the internal content of intrinsic elements of that cell. This could indicate the effects of the exposed metal on the physiology of cells of *S. cerevisiae*. Besides P, four other intrinsic elements (Mg, Cu, potassium (K), and Zn) are also chosen for this purpose.

**FIGURE 5:**
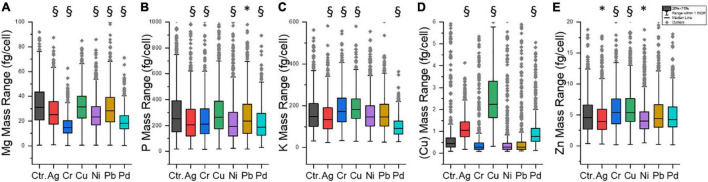
Content of five intrinsic elements at 10 ppm exposure of six different heavy metals. **(A)** Mg, **(B)** P, **(C)** K, **(D)** Cu, and **(E)** Zn. Ctr., control group. Each sample contained 491–893 individual cells (median: 766). * significantly different (*p* < 0.05). § significantly different (*p* < 0.005).

In fact, some differences were visible for the five elements depending on the metal exposure (Figure 5). When the heavy metal concentration in the P-excess medium was 10 ppm, the lowest mean Mg mass in the cells in the case of Cr was found, with a 50% decrease compared to control cells (Figure 5A). This may indicate that the growth of *S. cerevisiae* was reduced at 10 ppm of Cr, and this, in fact, agrees with literature findings: growth was inhibited by 30% at 0.78 ppm of Cr (Jianlong et al., 2004). However, it was also reported that *S. cerevisiae* would lose Mg during Cr uptake by bulk analysis (Zinicovscaia et al., 2020). SEM analyses have not shown significant changes in the cell surface of *S. cerevisiae* exposed to 10 ppm of Cr (Zinicovscaia et al., 2020). This is very consistent with the cell viability test in this study (Supplementary Table 4 and Supplementary Figure 5): 10 ppm Cr did not cause obvious cell death within 2 h of exposure.

In addition, Ag, Pd, and Ni had a slightly negative effect on the Mg mass (Figure 5A). In the case of Ni, it was reported to be taken up by *S. cerevisiae* through the Mg transport system (Joho et al., 1995), leading to a decrease in Mg content in cells through an uptake competition effect. These effects of the heavy metals on the growth of *S. cerevisiae* or specifically on its Mg content may be the reason why the correlations of metal mass and Mg mass (Figure 4) were not as strong as expected.

Figure 5B illustrates the distribution of P content in the tested cell populations. Ag, Cr, Ni, Pb, and Pd had significant effects on P content during P accumulation in yeast cells (*p* < 0.05 or 0.005). All median lines are lower than the control group. However, the P content did not change significantly at 10 ppm of Cu. These results reconfirmed that all heavy metals, except Cu, reduced the P content in the cell population of yeast during the P accumulation process.

Figures 5C–E shows that (I) Ag and Pb had limited effects on most of the investigated intrinsic elements. Metal exposure experiments on *S. cerevisiae* with higher concentrations were reported previously. For example, it was found that yeast cells lost their metabolic activity and proliferation ability during the incubation with 207 ppm of Pb (Van der Heggen et al., 2010); it was observed that the growth of yeast cells did not suspend under conditions of up to 216 ppm of Ag (Kierans et al., 1991). (II) Cu exposure had no remarkable effect on the content of other intrinsic elements except for Cu itself. (III) Pd made the average contents of P and K decrease, which may reflect Pd toxicity to the cells (Frazzoli et al., 2007). The SC-ICP-MS approach allows studying such effects also at lower exposure concentrations.

Multivariate analyses (principal component analysis, PCA; and canonical discriminant analysis, CDA) were performed to visually analyze the differences in the elemental content of the cell population with Mg, P, K, Cu, and Zn. It can be seen that at a concentration of 0.1 ppm, the data points of each group are mostly overlapped, and the 95% confidence ellipses are almost at the same position. This shows that 0.1 ppm, as well as 1 ppm of the heavy metals, had a limited influence on the elemental content in single cells (Supplementary Figure 6). However, when the concentration rose to 10 ppm, the data points and confidence circle from the Cu group (as exposure metal) shifted away from other groups.

**FIGURE 6:**
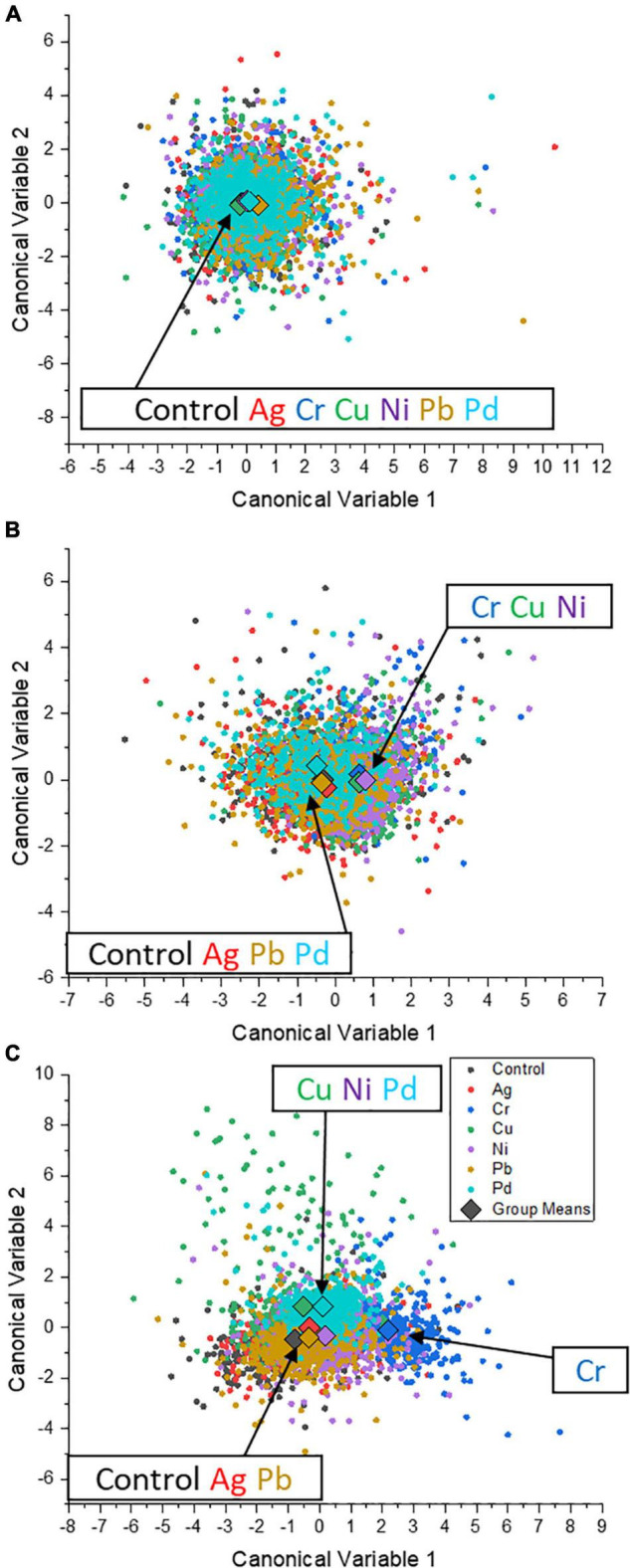
Canonical score plot of the canonical discriminant analysis for the exposure of six different heavy metals (Ag, Cu, Cr, Ni, Pb, and Pd) to *S. cerevisiae* cells during P feeding in P-excess medium. The exposure concentrations are 0.1 **(A)**, 1 **(B)**, and 10 ppm **(C)**. Group mean points are marked as diamonds in the graph.

More distinguishable details between heavy metals were confirmed by CDA: at 0.1 ppm, the data points in the coordinate system composed of canonical variables 1 and 2 mostly overlapped, while group means were close, proving a weak and limited influence on the physiological state of *S. cerevisiae* cells by the studied metal ions (Figure 6A). When the concentration was increased to 1 ppm, the group means of the groups Cr, Cu, and Ni tended to shift away from the control group (Figure 6B), suggesting a certain degree of effect on the intrinsic element profile in the cells of *S. cerevisiae* at 1 ppm. At 10 ppm, as shown in Figure 6C, the positions of each group were located differently compared with the coordinate position of the control group: close (groups Ag and Pb), middle (groups Cu, Ni, and Pd), and far (group Cr). It can be concluded that, in terms of intrinsic elements, Ag and Pb had the least impact on single cells of *S. cerevisiae*. Cu, Pd, and Ni had affected the composition of *S. cerevisiae* cells, while Cr had the largest influence as reflected by its shifted data points.

## Conclusion

The SC-ICP-TOF-MS was used to study the elemental contents of single yeast cells during P accumulation through intrinsic element information in the cells. This method can simultaneously obtain the information of multiple intrinsic and extrinsic elements in large numbers of single cells and, thus, determine elemental content distributions.

It was found that after the P starvation pretreatment, the yeast cells accumulated about 70% of P during the P feeding period. Exposure of the yeast cells to six heavy metals affected the P accumulation for most metals, especially at 10 ppm. This effect was strongest for Pd (−30% of P content), while Cu promoted the P uptake (+7% of P content). Furthermore, by comparing their cellular content and exposure concentration ratios, it was found that *S. cerevisiae* exhibited a higher uptake of Ag and Pd than Ni, Pb, and Cr. Unlike for other metals, yeast cells presented an active regulation for Cu content. At the same time, the influence of heavy metals on the intrinsic element composition of single cells was studied, and it was found that it was related to metal species, of which Cr had the greatest influence.

This study illustrates the potential of SC-ICP-TOF-MS to provide insight into the dynamic changes of metals and intrinsic elements of cells. It allows studying elemental uptake by single cells as well as the physiological reactions of cells upon exposure to toxic metals at the sublethal levels.

## Data Availability Statement

The raw data supporting the conclusions of this article will be made available by the authors, without undue reservation.

## Author Contributions

WQ performed the measurements, processed the experimental data, performed the analysis, drafted the manuscript, and designed the figures. All authors were involved in planning, discussed the results, and wrote the manuscript.

## Conflict of Interest

The authors declare that the research was conducted in the absence of any commercial or financial relationships that could be construed as a potential conflict of interest.

## Publisher’s Note

All claims expressed in this article are solely those of the authors and do not necessarily represent those of their affiliated organizations, or those of the publisher, the editors and the reviewers. Any product that may be evaluated in this article, or claim that may be made by its manufacturer, is not guaranteed or endorsed by the publisher.

## Footnotes

1
https://www.tofwerk.com/software/tofware/
