# Supplementary material for: Exploring the Extent of Phosphorus and Heavy Metal Uptake by Single Cells of Saccharomyces cerevisiae and Their Effects on Intrinsic Elements by SC-ICP-TOF-MS
Source: Front Microbiol. 2022 Apr 25;13:870931. doi: 10.3389/fmicb.2022.870931 (PMC9082303; doi:10.3389/fmicb.2022.870931)
Supplement: Supplementary file 1 [file Data_Sheet_1.docx]

# **Electronic Supplementary Material**

**Exploring the extent of phosphorus and heavy metal uptake by single cells of *Saccharomyces cerevisiae* and their effects on intrinsic elements by SC-ICP-TOF-MS**

Wen Qin^1^, Hans-Joachim Stärk^1^, Susann Müller^2^, Thorsten Reemtsma^1,3*^

^1^ Department of Analytical Chemistry, Helmholtz Centre for Environmental Research – UFZ, Permoserstrasse 15, 04318, Leipzig, Germany

^2^ Department of Environmental Microbiology, Helmholtz Centre for Environmental Research – UFZ, Permoserstrasse 15, 04318, Leipzig, Germany

^3^ Institute of Analytical Chemistry, University of Leipzig, Linnéstrasse 3, 04103, Leipzig, Germany

* Correspondence author

Table S1. The composition of medium for the cells. Quantity of each substance is given that is required for composing 1 liter medium. pH = 5.4 - 5.5.

| Substance | Quantity |
| --- | --- |
| Glucose | 30.09 g |
| (NH_4_)_2_SO_4_ | 4.49 g |
| (NH_4_)_2_HPO_4_ | 1.85 g (0.434 g L^-1^ P in medium)  0 g (0 g L^-1^ P in medium)  6.6 g (1.55 g L^-1^ P in medium) |
| Sodium citrate × 2 H_2_O | 6.62 g |
| MgSO_4_ × 7 H_2_O | 0.345 g |
| MnSO_4_ × H_2_O | 9 mg |
| ZnSO_4_ × 7 H_2_O | 8.9 mg |
| CuSO_4_ × 5 H_2_O | 2.5 mg |
| CaCl_2_ × 2 H_2_O | 0.426 g |
| KCl | 0.9 g |
| FeCl_3_ | 9.1 mg |
| Myo-inositol | 0.06 g |
| Calcium pantothenate | 0.06 g |
| Thiamine Hydrochloride | 6.1 mg |
| Pyridoxine hydrochloride | 1.5 mg |
| Biotin | 3 mg |

Table S2. Typical parameters of icpTOF for the SC-ICP-TOF-MS measurements

| Parameters | Values |
| --- | --- |
| Measure mode | Time-resolved |
| Plasma power/W | 1550 |
| Plasma cooling flow/(l min^-1^) | 14 |
| Nebulizer flow/(l min^-1^) | 1.0 |
| Sample inlet speed/(ml min^-1^) | 0.3 |
| Dwell time/s | 0.003 |
| Acquisition time/s | 60 |
| Target isotopes | 24Mg, 31P, 39K, 52Cr, 58Ni, 63Cu, 64Zn, 106Pd, 107Ag, 208Pb |

Table S3. The analyzed cell number for all cell samples in this experiment.

| Samples for P accumulation studies | Cell number | Samples of 0.1 ppm heavy metal exposures | Cell number | Samples of 0.1 ppm heavy metal exposures | Cell number | Samples of 0.1 ppm heavy metal exposures | Cell number |
| --- | --- | --- | --- | --- | --- | --- | --- |
| Cells in standard medium | 1147 | Control | 856 | Control | 1147 | Control | 808 |
| Cells in P excess medium | 459 | Ag | 873 | Ag | 973 | Ag | 893 |
| Cells in P free medium and P excess medium | 1033 | Cu | 943 | Cu | 427 | Cu | 610 |
|  |  | Cr | 939 | Cr | 470 | Cr | 686 |
|  |  | Ni | 823 | Ni | 488 | Ni | 845 |
|  |  | Pb | 921 | Pb | 846 | Pb | 822 |
|  |  | Pd | 904 | Pd | 779 | Pd | 537 |

Table S4. The results of cell viability analysis. Cell photo was made by optical microscope after methylenen blue staining. N=3.

| Heavy metal | Test 1 | | | Test 2 | | | Test 3 | | | Average |
| --- | --- | --- | --- | --- | --- | --- | --- | --- | --- | --- |
|  | Dead | Total | Viability | Dead | Total | Viability | Dead | Total | Viability | Viability rate |
| control | 1 | 99 | 0.99 | 0 | 134 | 1.00 | 0 | 128 | 1.00 | 1.00 |
| Pd | 3 | 95 | 0.97 | 1 | 104 | 0.99 | 4 | 77 | 0.95 | 0.97 |
| Cu | 2 | 131 | 0.98 | 2 | 125 | 0.98 | 0 | 135 | 1.00 | 0.99 |
| Cr | 0 | 82 | 1.00 | 0 | 95 | 1.00 | 1 | 102 | 0.99 | 1.00 |
| Pb | 1 | 129 | 0.99 | 1 | 114 | 0.99 | 0 | 112 | 1.00 | 0.99 |
| Ag | 1 | 125 | 0.99 | 1 | 118 | 0.99 | 4 | 110 | 0.96 | 0.98 |
| Ni | 0 | 127 | 1.00 | 1 | 107 | 0.99 | 1 | 132 | 0.99 | 0.99 |

Dead: the number of dead cell. Total: the total number of cell. Viability rate = 1 – (dead/total).

Figure S1. P content of single cells of S. cerevisiae after different treatments: control; P feeding; P feeding after P starvation. Outliers were defined as > 1.5 IQR.


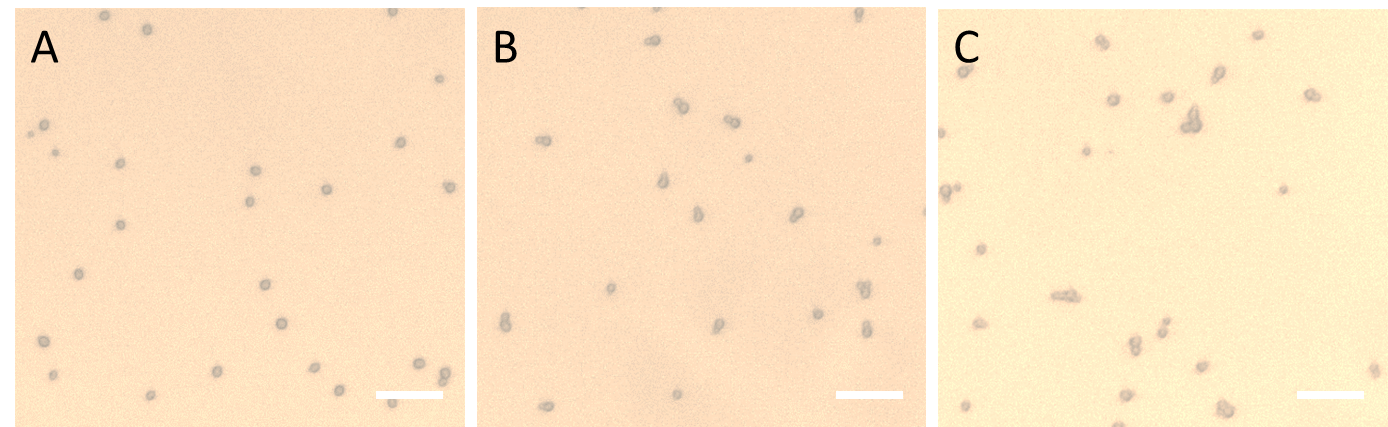


Figure S2. Cell microscopic photographs. Control cells (A), cells in P feeding medium after 2 h (B), and cells in P starvation medium for 4 h then in P feeding medium for 2 h (C). White bar is 50 um.

Figure S3. The percentage of cells with or without six target heavy metals in the cell population. Grey bar means that the single cell contains the content of target heavy metal; white bar indicates the opposite. Triplicate measurements were performed.

Figure S4. Elemental content of Cu (a) and cell percentage with Cu (b) under three different concentrations of Cu. Additional Cu as the heavy metal was added in P feeding medium in the concentrations of 0 ppm (control cells), 0.1 ppm, 1 ppm, and 10 ppm. **: significant difference to control samples, p < 0.05.


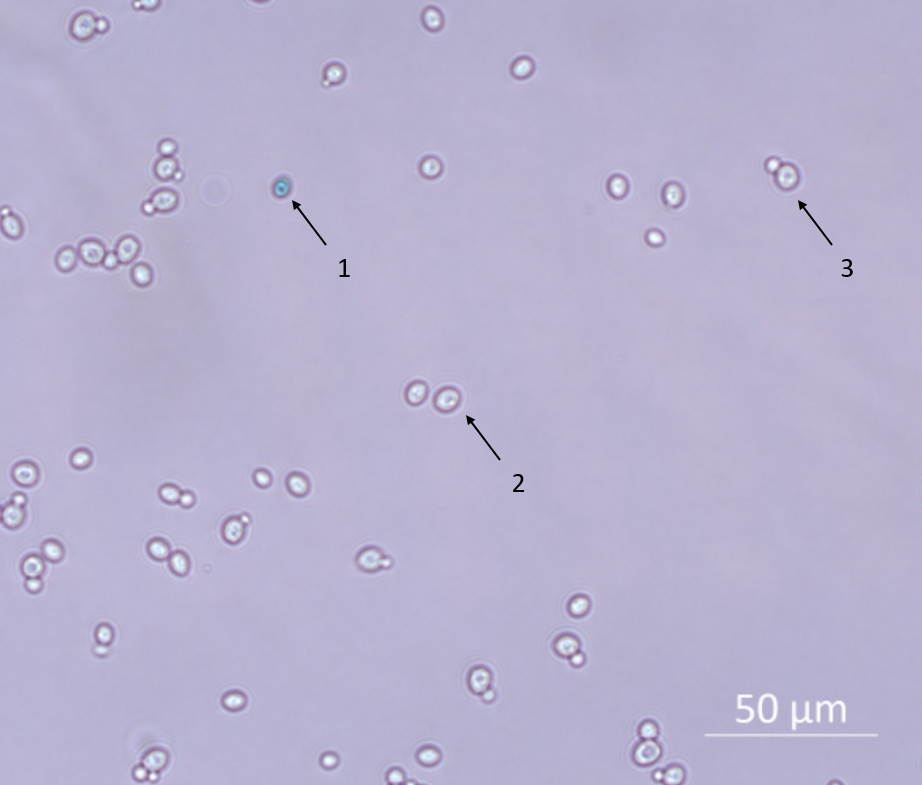


Figure S5. An example of cell viability test by methylene blue staining: dead cell (1), a live single cell (2) and a live budding cell (3).


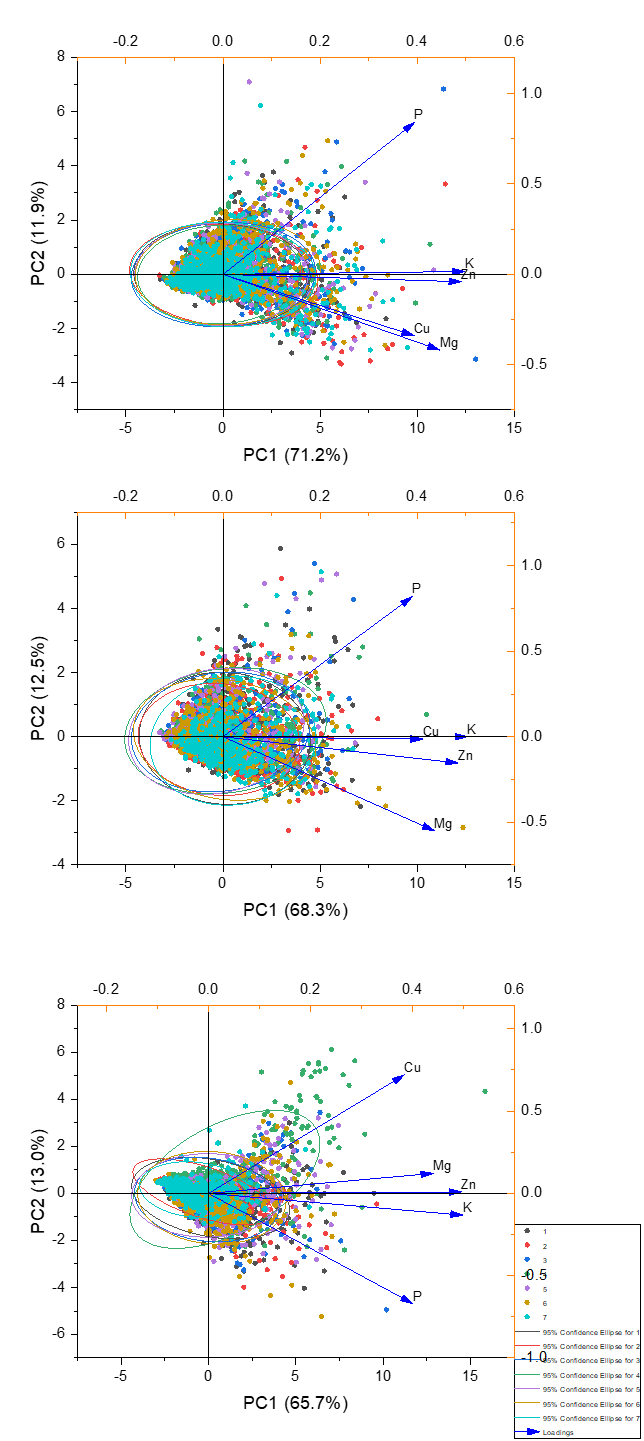


Figure S6. Score plot of the principal component analysis for the six different heavy metal exposures to S. cerevisiae cells during phosphorus feeding. Samples: Control (1), Ag (2), Cr (3), Cu (4), Ni (5), Pb (6), and Pd (7). The exposure concentrations are 0.1 ppm (top), 1 ppm (medium), and 10 ppm (bottom).
